# A Multimodal Stress-Prevention Program Supplemented by Telephone-Coaching Sessions to Reduce Perceived Stress among German Farmers: Results from a Randomized Controlled Trial

**DOI:** 10.3390/ijerph17249227

**Published:** 2020-12-10

**Authors:** Marita Stier-Jarmer, Cornelia Oberhauser, Dieter Frisch, Götz Berberich, Thomas Loew, Carina Schels-Klemens, Birgit Braun, Angela Schuh

**Affiliations:** 1 Chair of Public Health and Health Services Research, Institute for Medical Information Processing, Biometry, and Epidemiology (IBE), Ludwig-Maximilians-Universität München, 81377 Munich, Germany; coberhauser@ibe.med.uni-muenchen.de (C.O.); dfrisch@ibe.med.uni-muenchen.de (D.F.); angela.schuh@med.uni-muenchen.de (A.S.); 2 Pettenkofer School of Public Health, Institute for Medical Information Processing, Biometry and Epidemiology (IBE), Ludwig-Maximilians-Universität München, 81377 Munich, Germany; 3 Klinik für Psychosomatische Medizin und Psychotherapie, Windach, 86949 Windach, Germany; berberich@klinik-windach.de; 4 Department of Psychosomatic Medicine, University Hospital of Regensburg, 93053 Regensburg, Germany; thomas.loew@klinik.uni-regensburg.de (T.L.); birgit.braun@klinik.uni-regensburg.de (B.B.); 5 Department of Psychosomatic Medicine, Römerbad-Klinik GmbH/Klinik Kaiser Trajan, 93333 Bad Gögging, Germany; dr.schels_klemens@icloud.com

**Keywords:** mental health, agriculture, farmers, stress management, health resorts, balneotherapy, physical fitness

## Abstract

This study compared the effectiveness of a 12-day stress-prevention program (SGS) supplemented by individualized, structured, four-session telephone-coaching to that of an SGS without telephone-coaching in entrepreneurs from the green professions presenting with increased stress levels. All participants went through the SGS before being randomized either to the telephone-coaching group (TC) or to the control group without telephone-coaching (noTC). SGS included four key therapeutic elements: stress-management intervention, relaxation, physical exercise, and balneotherapy. The primary outcome was the current degree of subjectively experienced stress assessed with the Perceived Stress Questionnaire (PSQ) at a 9-month follow-up. Secondary outcomes included burnout symptoms, well-being, health status, sleep disorders, expectation of self-efficacy, depression, anxiety, ability to work, pain, and days of sick leave. Assessments were conducted at baseline, 12 days (end of program), and 1 (start telephone-coaching), 3, 6 (end of telephone-coaching), and 9 months. Data from 103 adults (TC = 51; noTC = 52), mostly fulltime farmers, were available for analysis (mean age: 55.3; 49.1% female). Participants experienced significant immediate improvement in all outcome measurements, which declined somewhat during the first three months after the end of SGS and then remained stable for at least another six months. While within-group changes from baseline to 9 months showed significant improvements at medium to large effect sizes for all target variables (PSQ-total, TC: −13.38 (±14.98); 95%-CI: (−17.68; −9.07); noTC: −11.09 (±14.15); 95%-CI: (−15.11; −7.07)), no statistically significant differences were found between the groups at any time and for any target variable (between-group ANCOVA for PSQ-total at 9 months, parameter estimator for the group: −1.58; 95%-CI: (−7.29; 4.13)). The stress-prevention program SGS is a feasible, effective, and practical way to reduce perceived stress and improve participants’ resources. Four subsequent telephone-coaching sessions do not seem to contribute to a further improvement in the results.

## 1. Introduction

Rising life expectancy, an increase in chronic illnesses, and longer working lives are among the challenges our social-security systems are increasingly facing. In particular, the prolonged working life requires timely strategies and measures to maintain working ability and earning capacity. A working world characterized by increasing time pressure, work overload, and growing complexity has also resulted in a change in work-related stress. In the past, physical stress was the main concern. Today there is an increase in psychosocial stress due to the above-mentioned changed working conditions.

We recently found the multimodal stress-prevention program “Im Moor zum inneren Gleichgewicht” (IMZIG) to be effective in reducing perceived stress and burnout symptoms in adults from different professions with above-average experienced stress and an increased risk of burnout in the short and medium terms [1]. The 3-week intervention, consisting of a stress-management seminar, relaxation techniques, physical activities, and peat baths, also provides the participants with self-help strategies to deal with everyday stressors in the medium and long terms. However, the 3-week absence from work required by the IMZIG program is not feasible for every professional group. Particularly in small (family) businesses, the 3-week absence of employer or employee to prevent health impairments may be difficult to explain. Therefore, the program has been reduced to two weeks to enable such professional groups to participate. Individualized, structured, four-session telephone-coaching was integrated into the follow-up to ensure the effectiveness of this abbreviated program.

A typical target group for such an abridged intervention could, for example, be participants in green professions, such as farming, forestry, and horticulture. Recent studies have shown that particularly farmers experience a considerable workload and lack of time [2]. International studies have shown that farmers are at an increased risk of mental health problems and experience elevated rates of psychological distress [3,4,5,6,7,8]. Studies on the mental well-being of self-employed persons report that, among the different types of self-employment, farmers are those with the poorest average mental well-being [9,10]. Stressors result from the nature of their profession and the context in which it is conducted [11]. Although most of these studies were carried out outside Europe, and no corresponding studies from German-speaking countries are available [8], most of the identified stressors are probably common to farmers around the world.

Working days in agriculture are long, and leisure is scarce. Agriculture is an economic sector in which work is performed predominantly by landowners and their family members. Farm families are characterized by the intertwining of work and family. Old and young live in close community; professional and private life are intensely interwoven. This offers opportunities for individual health, but also for potential conflicts and risks. Changes constantly challenge farmers and influence agricultural production, e.g., the uncertain market situation and resulting financial bottlenecks, restrictive agricultural legislation, technical innovations, required investments, and the unpredictable and often unstable or damaging weather conditions [2]. The most-cited risk factors affecting farmers’ mental health in developed countries, found by Yazd et al. in their systematic review of the relevant scientific literature published up to April 2019, included financial difficulties, pesticide exposure, weather uncertainty, poor physical health, government policies and regulations, heavy workloads, isolation, concerns about the future of the farm, working with family (role conflicts), and time pressure [8].

Our study’s stress-prevention program was supplemented by a process of individualized, structured, four-session telephone-coaching carried out during the follow-up to consolidate the effect of the 12-day stress-prevention intervention in the long term.

Telephone-coaching has been used for many years in the health sector, e.g., in the care, consultation, and aftercare of chronically ill patients [12,13,14,15]. It seems to be a viable and time-saving alternative to a two-person meeting if it aims at strengthening motivation and achieving certain health goals, such as changing an individual lifestyle. Coaching can motivate patients to live healthier lives by giving them concrete guidance on how to approach change and how to sustain healthy coping strategies. This increases self-confidence and conviction in the ability to help oneself.

A recent meta-analysis based on the results of 25 included studies on chronic heart failure indicated that tele-monitoring and structured telephone support can not only reduce hospital stays, but also improve the quality of life of those receiving support and, in some cases, even prevent death [13]. Reviews assessing telephone-coaching interventions in chronically ill patients show mainly positive results, indicating that telephone-coaching can positively influence the health behavior of affected patients, strengthen their self-efficacy, and improve their states of health [14,15].

In conclusion, previous research demonstrated that (a) the 3-week multimodal stress-prevention program IMZIG was effective in reducing perceived stress and burnout symptoms in adults from different professions with above-average experienced stress and an increased risk of burnout in the short and medium terms [1], and (b) telephone-coaching has been shown to be a feasible method of delivering behavioral change interventions [12,13,14,15]. The current study will (a) investigate the short- and medium-term effects of a stress-prevention program of similar content to IMZIG, but reduced to 12 days, and (b) assess whether the effects of the program can be strengthened and stabilized by telephone-coaching in the follow-up. The target group are entrepreneurs from the green professions (i.e., farmers, foresters and gardeners) exhibiting an increased level of psychological stress.

The overall objective of our research project was to develop, implement, and evaluate a 12-day program combining a stress-management intervention (SMI) with health resort treatment (HRT) supplemented by individualized, structured, four-session telephone-coaching. The 12-day stress-prevention program “Stark gegen Stress” (“Strong against Stress”; SGS), like its 3-week predecessor program IMZIG (1), aimed at reducing currently perceived stress, creating subjective resources, activating recovery processes for both body and mind, and providing strategies for dealing with stressors in everyday life.

Our hypotheses were that:

(1) participation in SGS would reduce perceived stress and improve further stress-related outcomes in the short and medium term;
(2) participants receiving SGS followed by telephone-coaching would have better outcomes compared to controls receiving SGS without subsequent telephone-coaching.

To test these hypotheses, we conducted a prospective, randomized, controlled study with a 9-month follow-up, comparing the stress-related outcomes of the intervention group (SGS followed by telephone-coaching) to those of a control group (SGS without subsequent telephone-coaching).

## 2. Methods

The study was designed as a dual-armed, randomized, controlled trial with assessments at baseline (T0), 12 days (T1; end of program), and 1-, 3-, 6-, and 9-month (T2-T5) follow-ups.

The inclusion criteria were as follows: (a) increased stress level and increased risk for developing physical and/or mental health impairments (confirmed by a physician); (b) membership in the Social Security for Agriculture, Forestry and Horticulture (SVLFG), retirement insurance; (c) 18+ years old; (d) actively working; and (e) a generally stable and at least moderately resilient physical and mental condition. Exclusion criteria were as follows: (a) acute infectious or immunosuppressive disease; (b) open wounds; (c) currently being treated for cancer; (d) mental and/or neurological disorders (e.g., moderate to severe depressive episode, anxiety disorder, dementia, epilepsy and other seizure disorders, acute relapses of multiple sclerosis); (e) acute and/or chronic advanced cardiovascular disease (e.g., arterial hypertension with hypertensive decompensation/cardiac crises, heart failure grade New York Heart Association - NYHA III-IV); and (f) acute and/or severe chronic diseases of the respiratory system (e.g., bronchial asthma with relapsing status asthmaticus, chronic obstructive pulmonary disease - COPD gold III and IV).

All eligible subjects participated in a 12-day prevention program including four key therapeutic elements:

(1) SMI: (10 × 2 h) (Table 1), each of the 10 sessions consists of a theoretical part and practical exercises;
(2) Relaxation: autogenic training (2 × 1 h), progressive muscle relaxation according to Jacobson (2 × 1 h), mindfulness-based stress reduction (4 × 1 h), massage (4 × 30 min);
(3) Physical exercise: back schooling (2 × 1 h), aqua fitness (4 × 1 h), endurance sports activities (4 × 1 h);
(4) Balneotherapy: 4 × peat or sulfur baths (20 min) followed by a resting period (20 min).

Participation in balneotherapy and exercise training could be suspended for health reasons.

The components of the stress-prevention program for farmers, gardeners and foresters are based on the aforementioned 3-week prevention program IMZIG [1]. The adapted version of the program used in the current study lasted just under two weeks and included the treatments available in Bad Gögging (e.g., peat and sulfur baths).

The program was carried out in the Römerbad Clinics in Bad Gögging from January to March 2018, October to December 2018, and January to March 2019 (eleven 12-day periods, each group containing eight to twelve participants).

The SMI and all exercise and relaxation courses were directed by experienced professionals.

After finishing the prevention program, participants were randomly assigned in a 1:1 ratio to either stress-prevention plus telephone-coaching (TC) or stress-prevention only (noTC) using permuted blocks of twelve. The TC group then received telephone-coaching over the course of six months (four approximately 20 min telephone calls, one, two, four and six months after the completion of the prevention program), while the noTC group received no further intervention. This means that the telephone-coaching sessions started at the 1-month follow-up and ended at the 6-month follow-up.

### 2.1. Telephone-Coaching

The objectives of the telephone-coaching were (1) the improvement of the quality of life, (2) psychoeducation, consolidation of knowledge from the seminar phase, (3) a review of the implementation of changes, (4) the analysis of barriers, (5) the promotion of motivation in the implementation of the intended changes, (6) the shortening, reduction, and avoidance of incapacity to work and inpatient stays and, if necessary, the arrangement of therapy places.

Procedure of the telephone-coaching: (1) identifying current stress symptoms and stress triggers, (2) repeating the goals and plans for change worked out in the seminar, (3) checking the changes made, (4) reinforcing the changes achieved, (5) analyzing the factors hindering change, (6) agreeing on new intermediate objectives, (7) supporting the structuring of everyday life, and (8) if necessary, motivating to seek further help (e.g., visiting a doctor, starting psychotherapy, complying with taking medication).

All participants documented their individual stress symptoms, goals, and change plans in structured, written records during the SMI. These records formed the basis for the telephone-coaching. As far as possible, telephone-coaching was always carried out by the same coach for one participant (relationship aspect).

### 2.2. Outcomes and Measures

The primary outcome was perceived stress (PSQ) at the 9-month follow-up (T5) [16,17,18]. Further standardized self-report instruments were used to measure (a) burnout symptoms (Maslach Burnout Inventory General Survey (MBI-GS-D)) [19], (b) well-being (World Health Organization 5-item Well-Being Index (WHO-5)) [20,21], (c) health status (EuroQol (EQ-5D-5L) general health index) [22,23], (d) sleep disorders (Insomnia Severity Index (ISI)) [24], (e) expectation of self-efficacy (Short Scale for Measuring General Self-Efficacy Beliefs (ASKU)) [25], (f) depression (Patient Health Questionnaire (PHQ-2)) [26], (g) anxiety (Generalized Anxiety Disorder (GAD-2)) [27], and (h) ability to work (Work Ability Index (WAI)) [28]. The frequency and intensity of bodily pain and its effects on everyday activities were also recorded. Participants’ satisfaction with the interventions received was assessed at the end of the 12-day stress-prevention program and after completion of the telephone-coaching using self-developed questionnaires.

### 2.3. Data Analysis

The sample size estimation was based on the PSQ-total and revealed a minimum of 96 participants, allowing for dropouts.

Missing items were substituted with the participants’ average of all non-missing items in the respective subscale. The recommendations of the authors of the questionnaire were applied where available. For the intention-to-treat (ITT) analysis performed for the PSQ sum score, the last observation was carried forward for dropouts or single missing time points. Effects were judged significant at *p* < 0.05 (two-sided) unless otherwise stated.

Baseline measurements and demographics were compared between groups (TC vs. noTC) using the independent samples t-test for metric and Pearson’s chi-square test for categorical variables. Changes from the baseline in primary and secondary outcomes after the end of the program and within the 9-month follow-up (i.e., the individual differences between one latter measurement and the measurement at baseline) were analyzed within groups using paired t-tests or Wilcoxon tests, and were compared between groups using unpaired t-tests or Mann–Whitney U tests and ANCOVA with adjustment for baseline values. Standardized effect sizes (Cohen’s *d*) were calculated as the difference between the two group means divided by the pooled standard deviation.

Statistical analyses were performed using the R version 3.6.1 [29] and IBM SPSS Statistics for Windows, Version 25.0 (IBM Corp., Armonk, NY, USA). 

Due to the nature of the interventions used, participants and therapists could not be blinded to the treatment received. The participants were partly blinded to the study hypothesis by receiving only partial information and were not informed about the main purpose of the study.

### 2.4. Compliance with Ethical Standards and Registration

All subjects gave their informed consent for inclusion before they participated in the study. The study was conducted in accordance with the Declaration of Helsinki, and the protocol was approved by the Ethics Committee of the LMU Munich, Germany (study no. 17–789).

The study was registered with the German Clinical Trials Register (DRKS-ID: DRKS00013771).

## 3. Results

### 3.1. Participant Flow

**Figure 1:**
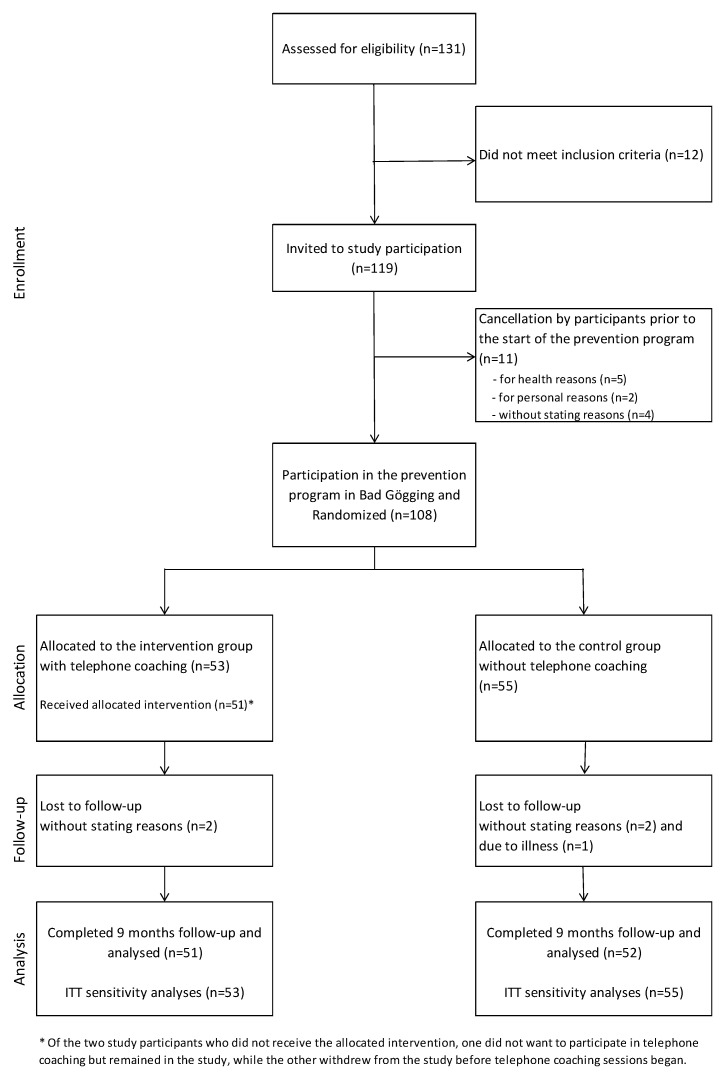
Participant flow.

Figure 1 shows the participant flow throughout the study. Eleven invited participants cancelled at short notice or did not show up in Bad Gögging. If a reason for non-participation was given, in most cases it was due to an acute illness of the participant or a family member. Of the 108 initially recruited participants, 5 were lost to follow-up. Data from 51 persons in the TC and 52 persons in the noTC group were available for analysis for the complete study period.

### 3.2. Participant Characteristics at Baseline

Table 2 shows the demographics and clinical variables of the final groups of participants. There were no statistically significant differences between groups at the baseline in terms of demographics and outcome variables considered.

The mean age was 55.3 years (±6.7). Almost half of the participants were female (49.1%). Participants came from almost all parts of Germany; 40.8% from the central and northern parts of the country and 59.2% from southern Germany. In total, 39.6% had no days of sick leave during that period. For those who had at least one day of sick leave, the mean number of days of sick leave during the previous six months was 16.6 (±26.2). Further, 73.1% were self-employed, mostly landowners (94.4%), 65.7% were engaged in animal husbandry, 62.8% in arable farming, 13.7% grew vegetables/special crops, and 7.8% operated a biogas plant. Every second farm (49.1%) employed external and/or seasonal workers. Regarding the future of their company over the next five years, 40.7% of the participants stated that they planned to maintain the current situation. One in three wanted to expand the business a little; one in ten wanted to significantly expand it. In total, 5% wanted to reduce their business, and two participants planned to give up their business completely.

The average PSQ-total at baseline was 55.3 (±14.6), which was significantly higher (i.e., worse) than in healthy adults with a mean of 33 [18]. One in four participants had a normal (25.9%; TC 18.9%; noTC 32.7%), one in three an increased (32.4%; TC 37.7%; noTC 27.3%), and more than 40% of the participants had a high (41.7%; TC 43.4%; noTC 40.0%) stress level, according to Kocalevent et al. [30]. Participants had a mean MBI-Emotional Exhaustion (MBI-EE) of 4.05 (±0.89). In total, 57.4% were at risk of burnout; 14.8% exhibited initial symptoms of a burnout syndrome. Among the psychological symptoms, 24.1% (PHQ-2) and 39.2% (GAD-2) of the participants had a sum score of 3 or higher, indicating that these individuals may have had symptoms of possible depression or anxiety, and further investigation of symptoms by a mental health professional may have been warranted. Almost 80% of the participants had latent (46.3%), moderate (27.8%), or severe (4.6%) sleep disorders; 60.8% of the participants described their current working ability as moderate, and 19.6% as critical.

### 3.3. Change in PSQ-Total and Secondary Outcomes

**Figure 2:**
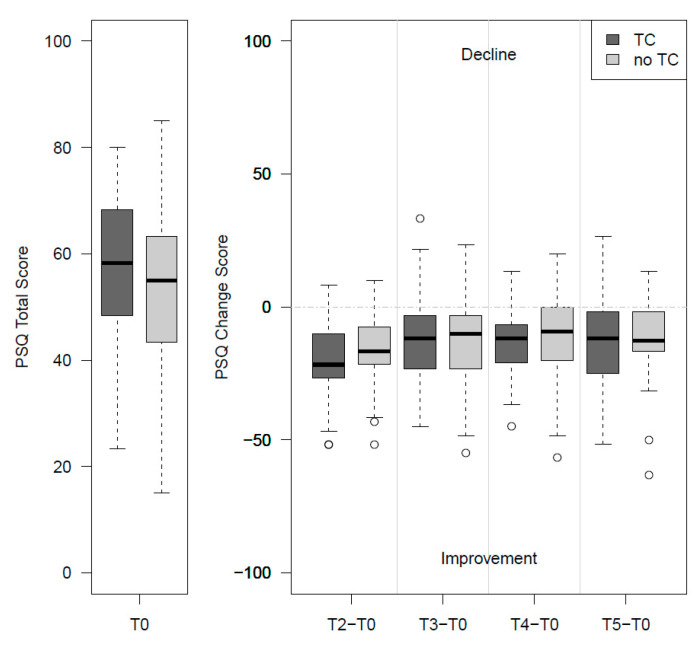
Perceived stress at baseline and change in score over time—TC (*n* = 51) versus noTC (*n* = 52). TC: telephone-coaching group; noTC: control group without telephone-coaching; PSQ: Perceived Stress Questionnaire; T0: baseline; T2, T3, T4, T5: 1-, 3-, 6-, and 9-month follow-up.

Results regarding changes over time in the PSQ-total and secondary outcomes compared between groups (TC vs. noTC) are presented in Table 3 and Figure 2. Table 4 shows the changes at the 9-month follow-up compared to baseline within the groups and for both groups combined. While within-group changes showed significant improvements at medium to large effect sizes for all target variables (Table 4), no statistically significant differences were found between the groups at any time and for any target variable (Table 3).

### 3.4. Within-Group Changes

At the 9-month follow-up, more than half of the participants (55.3%; TC 58.8%; noTC 51.9%) showed a normal stress level compared to 25.9% at baseline (Table 2). About one in four (26.2%; TC 21.6%; noTC 30.8%) revealed an increased stress level and almost one in five (18.4%; TC 19.6%; noTC 17.3%) a high stress level.

Classified according to Morin et al. [31], 27.8% of the study participants had a moderate and 4.6% a severe clinically significant insomnia at baseline (Table 2). After nine months, the proportion of participants with moderate insomnia had halved (13.6%), and only one participant still showed symptoms of severe insomnia. The initial mean ISI overall scores in both groups were markedly above the cut-off value of 10 points recommended in studies for the detection of insomnia [31,32]. After a significant improvement one month after participation in the prevention program, the values increased again slightly in both groups during the follow-up, but on average remained in the clinically unremarkable range at all measurement points.

While initially more than 90% of participants reported having had pain during the previous four weeks, about 60% of whom had experienced pain periodically, often, or very often, this figure was still just under 80% at the end of the 9-month follow-up. The most common complaint was back pain, followed by pain in the legs and knees and shoulders. Headaches were mentioned least frequently. The proportions of those who experienced pain periodically, often, or very often declined during the follow-up. The mean pain intensity was in the medium range at most of the measurement points and in the low range at T1. At baseline, about 60% of the participants reported that the pain they felt moderately, fairly, or very much affected their daily activities at home and at work. During follow-up, this rate levelled off at 40%.

The proportion of participants who had no days of sick leave in the previous six months increased considerably in both groups (T0_TC_ = 41.2%; T5_TC_ = 66.7%; T0_noTC_ = 38.2%; T5_noTC_ = 59.6%). Among study participants with at least one day of sick leave in the respective period, the average number of days of sick leave in the past six months increased significantly in both groups (M TC_T0_ = 14 ± 23; M TC_T5_ = 18 ± 48; M noTC_T0_ = 19 ± 29; M noTC_T5_ = 23 ± 37), while the median number decreased (MD TC_T0_ = 5; MD TC_T5_ = 3; MD noTC_T0_ = 10; MD noTC_T5_ = 5). The number of participants with long periods of illness (≥30 days) in the previous six months also decreased in both groups (N TC_T0_ = 5; N TC_T5_ = 2; N noTC_T0_ = 7; N noTC_T5_ = 5).

### 3.5. Participant Satisfaction

In general, participant assessments of the 12-day prevention program were clearly positive. Among the therapeutic interventions, balneotherapy and physical exercises in particular received (very) good ratings. In response to the question “What has the prevention program done for you?”, the three most frequently mentioned issues were (a) distance from everyday life and problems/finding peace/serenity/balance (41.3%), (b) not being alone with one’s problems/exchange with colleagues/openness in communicating with the group (32.7%), and c) relaxation/rest/recharging the batteries/well-being (31.7%). One participant was rather dissatisfied with the services received, but 85.8% were satisfied, and 13.2% rather satisfied with the therapeutic offer. In total, 91.4% of the participants considered the length of stay to be appropriate, and 6.7% found it too short; 93.4% stated they would participate in the program again.

The telephone-coaching was also rated quite positively overall. In addition, forty (80.0%) of the telephone-coaching participants used the opportunity to report on their experiences with the calls and the benefits they gained from them in more detail when asked “What has telephone-coaching done for you?” However, the evaluation of the telephone-coaching did not reveal a uniform trend. There were obviously participants who found it very positive, while others did not benefit from it.

## 4. Discussion

This study evaluates a 12-day stress-prevention program supplemented by individualized, structured, four-session telephone-coaching. The program combined the classical elements of health resort medicine (balneotherapy, relaxation, physical exercise) with an SMI, which mainly followed a psychoeducational approach. After participation in the 12-day program, study participants were randomly assigned to two groups. The intervention group (TC) received individual, structured, four-session telephone-coaching during the follow-up starting one month after the end of the program, while the control group (noTC) received no further intervention.

One month after participation in the 12-day stress-prevention program in Bad Gögging, there were remarkable statistically significant improvements in both groups in perceived stress, well-being, burnout symptoms, sleep disorders, psychological symptoms, general health status, and pain. These weakened somewhat during the 9-month follow-up. However, the comparison between baseline and the end of the study showed significant improvements in all target variables in both study groups even after nine months. The differences between groups were small and failed to be significant, which indicates that the telephone-coaching did not provide significant additional benefit. However, the telephone-coaching was rated predominantly as positive, but the participants involved obviously responded very differently to the offer. In particular, the answers to the question from the participant satisfaction survey “What has telephone-coaching done for you?” revealed great differences among the participants, with some benefiting greatly from telephone-coaching, while others were unable to make use of it.

Change over time in both groups yielded medium to large effect sizes, suggesting significant improvements in psychological functioning after participation in the 12-day stress-prevention program and over the course of the follow-up (Table 4).

The question of which specific interventional measures are essential for efficiently reducing stress was not investigated in the scope of this study. In previous studies, stress-reducing effects were demonstrated for most of the therapeutic measures applied in our stress-prevention program [33,34,35,36,37,38,39,40,41,42]. 

A comparison of the present results of the SGS with those of the corresponding IMZIG evaluation study in 2016 with a 6-month follow-up [1] reveals marked differences. Compared to the SGS, IMZIG participants were on average slightly younger (mean age IMZIG: 50.9 years; SGS: 55.3 years), predominantly female (proportion of women IMZIG: 76.1%; SGS: 49.1%), and practiced a wide range of different occupations in full-time (56.8%), part-time (19.3%), or self-employed (11.4%) capacities. There are also clear differences between the two study populations regarding the common primary and secondary outcomes. The primary outcome, subjectively perceived stress (PSQ-total), was significantly higher (i.e., worse) in IMZIG participants. While the mean of 68.4 points for IMZIG participants indicated a high stress level, SGS participants were in the range of an increased stress level with a mean of 55.3 points. Similar differences can be observed in well-being (WHO-5) (IMZIG: 30.7; SGS: 45.0), burnout risk (MBI-EE) (IMZIG: 4.5; SGS: 4.1), and general health (EQ5D-NRS) (IMZIG: 59.2; SGS: 67.1). For all outcomes, the average baseline values of the IMZIG study population were significantly worse compared to the SGS. However, one and six months after participation in one of the IMZIG or SGS prevention programs, no relevant differences could be discerned. The average values of subjectively perceived stress one month after the completion of the prevention program, which also corresponded to the start of the telephone-coaching session, were about 35 to 38 points in both study populations (in the normal range), as were the burnout risk (MBI-EE), with about 3.4 points and the WHO-5 values (IMZIG: 64.0; SGS: TC 65.4 and noTC 63.5). After six months, the average values of subjectively perceived stress in both study populations were approximately 40 to 43 points (in the normal range), as were those for the burnout risk (MBI-EE). The WHO-5 values also converged. Six months after participation in one of the IMZIG or SGS prevention programs, they were at approx. 55 to 57 points in both studies, which is significantly higher (i.e., better) than at the start of the respective studies, but still clearly below the gender- and age-group-specific norm values of the general population [43].

In view of the recognizably poorer initial values, the IMZIG participants improved much more during the 3-week stress-prevention program and in the subsequent 6-month follow-up than the participants in the SGS program during a comparable period. This may be for a variety of reasons. Perhaps a 3-week program is actually more effective than a 12-day program. The different setting, staffing, and spatial conditions could also have had an influence. The IMZIG participants also had more potential for improvement due to their poorer initial values.

In summary, a similar pattern is evident in both studies. The stress-related parameters improved considerably one month after the end of the program. After a slight deterioration in the 3-month follow-up, they remained unchanged until the end of the study.

The evaluation of the effectiveness of the SGS program was not the main objective of this study. The chosen study design presumed that all participants would participate in the program. There was no control group that did not participate in the program, making the evaluation of its effectiveness only possible to a limited extent. However, a comparison with the IMZIG data shows that similar effects were also achieved here.

The aim of the telephone-coaching sessions was to maintain the effect of the 12-day stress-prevention program in the long term. We had also assumed that a 12-day intervention, such as SGS, would not yield as good results as a similar one lasting 3 weeks, such as in the IMZIG. In this case, the TC group could possibly have achieved further improvements by participating in the telephone-coaching sessions. The fact that the participants in the SGS program, as well as those in the IMZIG program, achieved values in the normal range for subjectively perceived stress, burnout symptoms, and well-being one month after completion of their respective stress-prevention programs is one reason why no further improvements could be achieved by participating in subsequent telephone-coaching in the SGS study. Another reason is that even in the control group without telephone-coaching (noTC), the positive outcomes resulting from the stress-prevention program were maintained until the end of the follow-up.

The results of a recent scoping review of mental health outcomes and interventions among farming populations worldwide [44] show that (1) there is increasing interest in the topic, (2) stress was the most prevalent outcome of interest, accounting for 41.9% of included studies, and (3) only few evaluations of mental health interventions/services have been published to date. The authors point to two common findings of these studies that aimed to assess interventions. One finding suggests that farmers need specifically tailored services provided by professionals who have a basic understanding of the specific pressures in agriculture. This coincides with the observations from our study, where the topic was repeatedly raised by the participants and some explicitly expressed the desire for a coach who has a knowledge base of agriculture. The second finding is that women were more willing to seek help for their mental health concerns than men. This is consistent with research on occupational stress, where study populations are often predominantly female [1,45]. In our current study, however, almost as many women as men were interested in and participated in the stress-prevention intervention.

### Limitations and Strengths

Some limitations of our study need to be addressed. (1) The specific effects can hardly be separated from the non-specific effects achieved when staying at a health resort. In a complex program, such as SGS, a multitude of therapeutic stimuli are contrasted with a multitude of therapeutically desired effects. These include the specific effects of the applied therapies, as well as the unspecific effects of the environment and setting (such as empathy shown by caregivers who are actively involved in an intervention accepted by the ‘patient’). Research on psychotherapeutic interventions has demonstrated that non-specific effects can significantly contribute to the overall effect of interventions [46]. (2) The study sample used was a convenience sample, which is limited in generalizability and representativeness. (3) Further limitations to this study comprise the potential bias of the self-report, much like the social-desirability bias [47].The question arises of to what extent the participants (all insured persons of the SVLFG, which financed the entire program) felt committed to the intervention program and may have responded in a socially desirable way. (4) It also cannot be excluded that the results could be biased due to the lack of blinding, as the blinding of participants and psychologists was not possible in this type of intervention. However, since the participants were uninformed about the exact research question, they were at least partly blinded.

The strengths of the study include its timeliness and relevance. As far as we know, there have not yet been any German studies on farmers’ psychological stress. The additional strengths are the randomized design, the use of validated, commonly accepted instruments, the high response rate, the low dropout rate, and the 9-month follow-up, which is not common in studies in this field [45,48].

## 5. Conclusions

In summary, our results using the example of German farmers show that the 12-day multimodal stress-prevention program “Stark gegen Stress” can effectively reduce the extent and perception of perceived stress over a period of at least nine months in people with increased stress levels and an associated risk of developing physical and mental health problems. The other effects observed include an improvement in the quality of sleep, well-being, quality of life, and the general state of health, as well as a reduction in psychological symptoms, such as depression or anxiety. Four subsequent telephone-coaching sessions did not seem to contribute to a further improvement of the results. Our results suggest that, despite its shortened duration, the 12-day stress-prevention program is a feasible, effective, and practical way to reduce perceived stress and improve participants’ resources. Due to the encouraging results of the study, the SGS has been implemented several times in early 2020. Subsequent telephone-coaching was not compulsory, but was offered and could be taken up by interested participants. The next steps in further research could be to clarify which types of interventions in the stress-prevention programs are most effective, and what are the characteristics of persons who find them beneficial.

## Figures and Tables

**Table 1:** Stress-management intervention (SMI)—Psychoeducation and psychological coaching—the contents in 10 key points.

(1) Of Good and Bad Stress
Introduction to the stress concept, eustress/distress, stress consequences, burnout. Mindfulness exercise.
(2) Detect Your Own Stress Signals
Warning signals for stress, development of individual stress symptoms, personal experiences, and previous coping options. Mindfulness exercises.
(3) Conditions and Behavior
External stressors (conditions) and inner drivers (basics of one’s own behavior). Mindfulness exercises.
(4) Body and Stress
Hormones and neurobiology, physical sequelae, physical protective mechanisms, sport, and stress. Mindful walk.
(5) Personality and Stress
Inner drivers and rational alternatives, own stumbling blocks, performance, perfectionism, and demarcation. Mindfulness exercise.
(6) Goals and Values
“Higher, faster, further … “, the cake of importance, personal goals. Mindfulness exercise.
(7) Stress Management 1
Basics: Sleep, regeneration, pleasure, biorhythm. Pleasure training.
(8) Stress Management 2
Setting limits, task management, delegating. Role-play.
(9) Stress Management 3
Leadership behavior, empathy and demarcation, cooperation, and responsibility. Role-play.
(10) I will change something
Transfer into everyday life: concrete definition of goals, points of change, time structure. Mindfulness exercise

**Table 2:** Baseline demographic and clinical characteristics.

Demographic and Clinical Characteristics	TC	noTC	Total
	(*n* = 53)	(*n* = 55)	(*n* = 108)
Age (years), M (SD)	54.4 (7.6)	56.1 (5.5)	55.3 (6.7)
Female, *n* (%)	25 (47.2%)	28 (50.9%)	53 (49.1%)
Marital status, *n* (%)			
married/cohabiting	48 (90.6%)	50 (90.9%)	98 (90.7%)
divorced/widowed	2 (3.8%)	4 (7.3%)	6 (5.6%)
single	3 (5.7%)	1 (1.8%)	4 (3.7%)
Highest educational level, *n* (%)			
general secondary school certificate	17 (32.1%)	18 (32.7%)	35 (32.4%)
intermediate secondary school certificate	19 (35.9%)	23 (41.8%)	42 (38.9%)
qualification for university/universities of applied sciences entrance	7 (13.2%)	5 (9.1%)	12 (11.1%)
university degree/universities of applied sciences degree	8 (15.1%)	6 (10.9%)	14 (13.0%)
other	2 (3.8%)	3 (5.5%)	5 (4.6%)
No days of sick leave (last 6 months), *n* (%)	21 (41.2%)	21 (38.2%)	42 (39.6%)
Number of days of sick leave (last 6 months), M (SD), MD	14.4 (22.9), 4.5	18.7 (29.0), 10	16.6 (26.2), 6
PSQ, M (SD)			
Worries	47.4 (19.2)	43.1 (17.5)	45.2 (18.4)
Tension	61.3 (18.1)	58.4 (18.9)	59.8 (18.5)
Joy	42.6 (14.3)	47.3 (19.4)	45.0 (17.1)
Demands	61.8 (18.3)	59.8 (19.9)	60.8 (19.1)
Total	57.1 (13.3)	53.5 (15.7)	55.3 (14.6)
PSQ, *n* (%)			
Stress level normal (≤45)	10 (18.9%)	18 (32.7%)	28 (25.9%)
Stress level increased (46–59)	20 (37.7%)	15 (27.3%)	35 (32.4%)
Stress level high (≥60)	23 (43.4%)	22 (40.0%)	45 (41.7%)
ISI, M (SD)			
Total	12.1 (5.0)	12.3 (6.0)	12.2 (5.5)
ISI, *n* (%)			
Absence of insomnia (<8 points)	10 (18.9%)	13 (23.6%)	23 (21.3%)
Sub-threshold insomnia (8–14 points)	25 (47.2%)	25 (45.5%)	50 (46.3%)
Moderate insomnia (15–21 points)	18 (34.0%)	12 (21.8%)	30 (27.8%)
Severe insomnia (>21 points)	0 (0.00%)	5 (9.1%)	5 (4.6%)
WHO-5, M (SD)			
Total	45.4 (18.7)	44.6 (19.6)	45.0 (19.1)
EQ-5D, M (SD)			
Health Status	67.1 (16.8)	67.1 (15.6)	67.1 (16.1)
EQ-5D, *n* (%)			
Mobility. no problems	35 (66.0%)	34 (61.8%)	69 (63.9%)
Self-care. no problems	49 (92.5%)	54 (98.2%)	103 (95.4%)
Usual activities. no problems	27 (50.9%)	31 (57.4%)	58 (54.2%)
Pain/discomfort. no problems	3 (5.7%)	5 (9.1%)	8 (7.4%)
Anxiety/depression. no problems	10 (18.9%)	22 (40.0%)	32 (29.6%)
MBI-EE, M (SD)			
Emotional Exhaustion	4.1 (0.8)	4.1 (1.0)	4.1 (0.9)
MBI-EE, *n* (%)			
No burnout (<3.6 points)	18 (34.0%)	12 (21.8%)	30 (27.8%)
Risk of burnout (3.6–5.0 points)	29 (54.7%)	33 (60.0%)	62 (57.4%)
Symptoms of burnout (>5.0 points)	6 (11.3%)	10 (18.2%)	16 (14.8%)
ASKU, M (SD)			
Total	3.6 (0.6)	3.8 (0.6)	3.7 (0.6)
PHQ-2, M (SD)			
Total	2.0 (1.3)	2.0 (1.0)	2.0 (1.1)
GAD-2, M (SD)			
Total	2.7 (1.6)	2.3 (1.3)	2.5 (1.5)
Pain frequency, *n* (%)			
none/now and then	19 (38.0%)	16 (32.0%)	35 (35.0%)
periodically/often/very often or permanently	31 (62.0%)	34 (68.0%)	65 (65.0%)
Pain intensity, M (SD), MD	3.5 (1.9), 3	4.3 (2.2), 4	3.9 (2.1), 4
Pain-related limitations in everyday life, *n* (%)			
Not at all	4 (7.7%)	6 (11.1%)	10 (9.4%)
Somewhat	18 (34.6%)	14 (25.9%)	32 (29.6%)
Moderate	19 (36.5%)	20 (37.0%)	39 (36.8%)
Considerable	10 (19.2%)	12 (22.2%)	22 (20.8%)
Severe	1 (1.9%)	2 (3.7%)	3 (2.8%)
WAI, M (SD)			
Total	32.3 (5.9)	31.5 (5.0)	31.9 (5.5)
WAI current work ability, *n* (%)			
Very good (>43 points)	0 (0.0%)	0 (0.0%)	0 (0.0%)
Good (37–43 points)	12 (24.0%)	8 (15.4%)	20 (19.6%)
Moderate (28–36 points)	29 (58.0%)	33 (63.5%)	62 (60.8%)
Critical (<28 points)	9 (18.0%)	11 (21.2%)	20 (19.6%)

PSQ: Perceived Stress Questionnaire; ISI: Insomnia Severity Index; WHO-5: World Health Organization 5-item Well-Being Index; EQ-5D: EuroQol (EQ-5D-5L) general health index; MBI-EE: Maslach Burnout Inventory, Emotional Exhaustion; ASKU: Short Scale for Measuring General Self-Efficacy Beliefs; PHQ-2: Patient Health Questionnaire 2-item; GAD-2: Generalized Anxiety Disorder 2-item; WAI: Work Ability Index; M: mean; SD: standard deviation; MD: median.

**Table 3:** Changes in outcomes from baseline to 1-, 6- and 9-month post-intervention follow-up (PP analysis, *N* = 103 at 9 months) and ITT analysis for the primary outcome PSQ-total considering all randomized persons (participants *N* = 103 and drop outs *N* = 5) with missing-value imputation using the “Last Observation Carried Forward” method.

	Intervention Group (TC)			Control Group (noTC)						
		Change from Baseline			Change from Baseline		Change from Baseline—Difference between Groups		Effect Size	Between-Group ANCOVA ^d^
	*N*	M	SD	*N*	M	SD	M [95% CI]	Pooled SD	Cohen’s *d*	Parameter Estimate for Group [95% CI]
PSQ-total (PP)										
1 month ^a^	49	−19.06	14.32	51	−16.06	12.62	−3.00 [−8.36; 2.37]	13.48	−0.22	−2.03 [−7.10; 3.03]
6 months ^b^	48	−13.27	12.19	50	−11.57	15.85	−1.70 [−7.37; 3.95]	14.18	−0.12	−0.98 [−6.50; 4.55]
9 months ^b^	49	−13.38	14.98	50	−11.09	14.15	−2.29 [−8.10; 3.53]	14.57	−0.16	−1.58 [−7.29; 4.13]
PSQ-total (ITT)										
1 month	53	−18.56	14.44	55	−14.81	12.99	−3.75 [−9.00; 1.50]	13.72	−0.27	−3.00 [−7.99; 1.99]
6 months	53	−12.54	11.91	55	−10.55	15.53	−1.99 [−7.26; 3.28]	13.87	−0.14	−1.09 [−6.47; 4.29]
9 months	53	−13.63	15.04	55	−10.33	13.74	−3.30 [−8.80; 2.21]	14.39	−0.23	−2.43 [−8.01; 3.15]
ISI (PP)										
1 month ^a^	51	−5.41	4.56	53	−4.20	4.78	−1.21 [−3.03; 0.60]	4.67	−0.26	−1.38 [−2.94; 0.18]
6 months ^b^	50	−3.81	5.27	52	−4.53	5.24	0.72 [−1.34; 2.79]	5.25	0.14	0.49 [−1.32; 2.30]
9 months ^b^	51	−2.97	4.84	52	−4.05	5.15	1.08 [−0.87; 3.04]	5.00	0.22	0.88 [−0.88; 2.64]
WHO-5 (PP)										
1 month ^a^	51	20.00	21.67	53	18.94	18.63	1.06 [−6.82; 8.93]	20.18	0.05	1.44 [−5.35; 8.23]
6 months ^b^	51	11.45	18.63	52	15.77	19.97	−4.32 [−11.86; 3.23]	19.32	−0.22	−3.75 [−10.41; 2.91]
9 months ^b^	51	11.69	17.74	52	12.77	18.98	−1.08 [−8.26; 6.10]	18.38	−0.06	−0.74 [−7.61; 6.14]
EQ5D Health Status (PP)										
1 month ^a^	51	10.76	16.52	51	11.88	14.24	−1.12 [−7.18; 4.94]	15.42	−0.07	−1.30 [−6.48; 3.88]
6 months ^b^	51	11.24	14.95	51	9.37	14.82	1.87 [−3.99; 7.71]	14.89	0.13	2.13 [−2.80; 7.05]
9 months ^b^	51	7.90	15.25	51	9.04	15.25	−1.14 [−7.13; 4.86]	15.25	−0.07	−0.86 [−5.90; 4.17]
MBI-EE (PP)										
1 month ^a^	51	−0.64	0.80	53	−0.69	0.89	0.05 [−0.29; 0.37]	0.85	0.06	0.03 [−0.27; 0.34]
6 months ^b^	51	−0.56	0.84	51	−0.49	1.00	−0.07 [−0.43; 0.29]	0.92	−0.08	−0.08 [−0.42; 0.26]
9 months ^b^	51	−0.51	0.80	52	−0.57	0.84	0.06 [−0.26; 0.38]	0.82	0.07	0.06 [−0.25; 0.37]
ASKU (PP)										
1 month ^a^	50	0.17	0.61	53	0.26	0.56	−0.09 [−0.31; 0.14]	0.58	−0.16	−0.22 [−0.41; −0.03]
6 months ^b^	50	0.27	0.57	51	0.24	0.55	0.03 [−0.19; 0.25]	0.56	0.05	−0.07 [−0.26; 0.12]
9 months ^b^	50	0.23	0.66	52	0.22	0.50	0.01 [−0.22; 0.24]	0.58	0.02	−0.08 [−0.28; 0.13]
PHQ-2 (PP)										
1 month ^a^	51	−0.67	1.18	53	−0.70	0.99	0.03 [−0.39; 0.46]	1.09	0.03	0.05 [−0.33; 0.42]
6 months ^b^	51	−0.29	1.17	52	−0.40	1.27	0.11 [−0.37; 0.59]	1.22	0.09	0.09 [−0.33; 0.50]
9 months ^b^	51	−0.41	1.06	52	−0.48	1.16	0.07 [−0.37; 0.50]	1.11	0.06	0.05 [−0.34; 0.44]
GAD-2 (PP)										
1 month ^a^	51	−1.08	1.43	52	−0.94	1.38	−0.14 [−0.68; 0.41]	1.40	−0.10	0.08 [−0.38; 0.55]
6 months ^b^	51	−0.84	1.51	51	−0.84	1.41	0.00 [−0.57; 0.57]	1.46	0.00	0.22 [−0.25; 0.68]
9 months ^b^	50	−1.02	1.45	51	−0.80	1.34	−0.22 [−0.77; 0.34]	1.40	−0.16	−0.00 [−0.44; 0.42]
WAI (PP) ^c^										
9 months ^b^	48	2.66	4.17	50	3.29	4.50	−0.63 [−2.37; 1.11]	4.34	−0.15	−0.45 [−2.19; 1.28]

PP, “per protocol”; ITT, “intention to treat”; N, number of cases; M, mean; SD, standard deviation; 95% CI, 95% confidence interval ^a^*N* = 105; ^b^ *N* = 103; ^c^ The Work Ability Index (WAI) was only used at the beginning (T0) and end (T5) of the study; ^d^ Between-group ANCOVA adjusted for baseline values.

**Table 4:** Change over time within the total group and within TC and noTC groups—mean differences at 9-month follow-up compared to baseline (t-test and effect sizes for paired samples).

	Group	Change from Baseline			Paired t-Test		Effect Size
		*N*	M	SD	[95% CI]	*p*-Value	Cohen’s d
PSQ-Total	Total	99	−12.22	14.54	[−5.71; −3.88]	<0.0001	0.84
	TC	49	−13.38	14.98	[−17.68; −9.07]	<0.0001	0.89
	noTC	50	−11.09	14.15	[−15.11; −7.07]	<0.0001	0.78
ISI	Total	103	−3.52	5.00	[−4.50; −2.54]	<0.0001	0.70
	TC	51	−2.97	4.84	[−4.33; −1.61]	0.0001	0.61
	noTC	52	−4.05	5.15	[−5.49; −2.62]	<0.0001	0.79
WHO-5	Total	103	12.23	18.29	[8.66; 15.81]	<0.0001	0.67
	TC	51	11.69	17.74	[6.70; 16.68]	<0.0001	0.66
	noTC	52	12.77	18.98	[7.49; 18.05]	<0.0001	0.67
EQ5D Health Status	Total	102	8.47	15.19	[5.49; 11.45]	0.0005	0.56
	TC	51	7.90	15.25	[3.61; 12.19]	<0.0010	0.52
	noTC	51	9.04	15.25	[4.75; 13.33]	0.0001	0.59
MBI-EE	Total	103	−0.54	0.82	[−0.70; −0.38]	<0.0001	0.66
	TC	51	−0.51	0.80	[−0.74; −0.29]	<0.0001	0.64
	noTC	52	−0.57	0.84	[−0.81; −0.34]	<0.0001	0.68
ASKU	Total	102	0.23	0.58	[0.11; 0.34]	0.0001	0.40
	TC	50	0.23	0.66	[0.04; 0.42]	0.0164	0.35
	noTC	52	0.22	0.50	[0.09; 0.36]	0.0020	0.44
PHQ-2	Total	103	−0.45	1.11	[−0.66; −0.23]	0.0001	0.41
	TC	51	−0.41	1.06	[−0.71; −0.11]	0.0078	0.39
	noTC	52	−0.48	1.16	[−0.80; −0.16]	0.0044	0.41
GAD-2	Total	101	−0.91	1.39	[−1.19; −0.64]	<0.0001	0.66
	TC	50	−1.02	1.45	[−1.43; −0.61]	<0.0001	0.70
	noTC	51	−0.80	1.34	[−1.18; −0.43]	0.0001	0.60
WAI	Total	98	2.98	4.33	[2.11; 3.85]	<0.0001	0.69
	TC	48	2.66	4.17	[1.44; 3.87]	0.0001	0.64
	noTC	50	3.29	4.50	[2.01; 4.57]	<0.0001	0.73

*N*: number of cases; M: mean; SD: standard deviation; 95% CI: 95% confidence interval.
